# Impact of Volcanic Slag on Cucumber Yield, Quality, and Rhizosphere Soil Environment

**DOI:** 10.3390/plants14091328

**Published:** 2025-04-28

**Authors:** Qi Chen, Xiaohong Li, Wanwu Zhang, Dongxu Xue, Qiyuan Sun, Hangtao Xing, Wei Wang, Chunyan Wu

**Affiliations:** 1 College of Horticulture, Jilin Agricultural University, Changchun 130118, China; 15143453656@163.com (Q.C.); x2693963339@163.com (D.X.); sunqiyuan0825@163.com (Q.S.); xinghangtao2024@163.com (H.X.); 2 College of Agriculture, Liaodong University, Dandong 118003, China; 15942527070@163.com; 3 Jilin Provincial Rural Economic Information Center, Changchun 130012, China; zhangwanwu2025@163.com

**Keywords:** cucumber, quality, soil environment, volcanic slag, yield

## Abstract

This study aimed to examine the effect of adding volcanic slag to soil on the growth, yield, and quality of cucumbers. It also analyzed the changes in the physicochemical properties of the rhizosphere soil, as well as the diversity and structural changes of the bacterial community present in the soil of the cucumber plants. This study used conventional fertilization and cultivation techniques and set up five treatments: HS500, HS1000, HS1500, and HS2000 (representing 500, 1000, 1500, and 2000 kg/ha of volcanic slag added per 667 sq.m in the cultivation trough, respectively), and control (CK; representing 0 kg of added volcanic slag). The Illumina MiSeq System was used to analyze the soil microbial community. The findings revealed that the HS1000 treatment had the most significant promoting effect on increasing cucumber yield, whereas the HS2000 treatment exhibited no significant change compared with the CK treatment. The HS500, HS1000, and HS1500 treatments increased the yield by 12.89%, 24.28%, and 19.56%, respectively, compared with the CK treatment. The HS1000 treatment increased the soluble sugar, vitamin C, and soluble solid contents by 12.39%, 17.57%, and 24.33%, respectively, compared with the CK treatment. The organic matter, total nitrogen, alkali-hydrolyzable nitrogen, nitrate nitrogen, ammonium nitrogen (NH4+-N), available potassium (AK), and available phosphorus (AP) contents in the rhizosphere soil of cucumber plants were the highest under the HS1000 treatment. The alpha diversity analysis revealed that the Chao1, Shannon, and ACE indexes reached the highest under the HS1000 treatment, which were significantly higher than the CK treatment. In contrast, the Simpson index and coverage had no significant changes between treatments. The dominant phyla in each treatment were Proteobacteria, Actinobacteria, and Acidobacteria, among others. The redundancy analysis of soil physicochemical properties and 15 bacterial genera of interest revealed that the available phosphorus, available potassium, and NH4+-N contents were the primary factors influencing the bacterial community in cucumber rhizosphere soil.

## 1. Introduction

Cucumber (*Cucumis sativus* L.) is an annual climbing plant belonging to the gourd family [1]. Cucumbers are a popular vegetable, rich in various vitamins and minerals that are beneficial to the human body. However, long-term, high-intensity, continuous intensive cucumber planting and unreasonable use of pesticides and fertilizers in facilities lead to a significant depletion of soil nutrients, alteration of soil physical and chemical properties, disruption of beneficial soil bacterial community, decrease in the soil bacterial diversity, a significant reduction in beneficial bacterial species, and promotion of pathogenic bacteria. This results in severe soil acidification and salinization, causing a decrease in cucumber yield and quality in facility cultivation [2,3].

Volcanic slag is a type of basic magma usually formed when magma is ejected from a narrow volcanic channel onto the ground, which is then cooled after high-temperature combustion and ejection, forming a slag-like volcanic gravel [4]. Volcanic slag has a complex composition, mainly comprising volcanic glass and a small proportion of zeolite [5]. The main mineral components of volcanic slag are 29.48% quartz, 30.04% alkaline feldspar, and 40.06% plagioclase [6], containing minerals such as silicon dioxide, calcium, phosphorus, sodium, and potassium. Qian Cheng et al. [7] also found trace elements such as iron, magnesium, titanium, lead, and barium in volcanic slag. Volcanic slag has good water permeability and water retention capacity, which helps improve soil properties by enhancing ion exchange and selective exchange capabilities [8]. Besides containing the necessary calcium, silicon, nitrogen, phosphorus, potassium, and various trace elements for soil, volcanic slag has a promoting effect on the physical and chemical properties of soil, such as acidity and alkalinity, ion exchange capacity, and release of soil nutrients, due to its special physical properties.

A significant body of research has shown that volcanic slag can be used as a substrate for vegetable growth. Combined with a reasonable topdressing method, it promotes the growth and development of vegetable crops such as cabbage [9], carrots [10], lettuce [11], eggplant [12], spinach [13], melon [14], and celery [15]. It also significantly increases the protein, calcium, and vitamin C contents [16]. Moreover, volcanic slag serves as a conditioner, which helps increase the organic matter, alkali-released nitrogen, quick-acting phosphorus, and quick-acting potassium contents in pepper soil [17]. At the same time, studies have demonstrated that the application of volcanic ash has a significant mitigating effect on the saline-alkali stress experienced by melons [18].

Recent studies have found that applying soil conditioners can affect the structure and quantity of soil microbial communities. A study indicated that treating cadmium-contaminated soil with garlic and soil conditioners leads to a significantly increased abundance of Proteobacteria in acidic soil and Actinobacteria in alkaline soil [19]. Through a study of the effects of wood vinegar on tomato rhizosphere soil, it was found that the application of wood vinegar could enhance soil nutrition and increase the abundance of beneficial microorganisms. This suggests significant potential for improving the microecological environment of rhizosphere soil [20].

Adding soil conditioners can enhance the Chao1 index of oilseed rape acidic soil microorganisms, significantly increasing the abundance of Subgroup-6, Lysobacter, Massilia, and Ramlibacter relative to other microorganisms. They also improve the Shannon and Simpson diversity indexes of the bacterial community in pepper continuous cropping soil, as well as the ACE and Chao1 richness indexes [21]. At the same time, soil conditioners also reduce the Shannon and Simpson diversity indexes of the fungal community, as well as the ACE and Chao1 richness indexes. These changes alter the dominant phyla in the soil Firmicutes, Proteobacteria, Bacteroidetes, and Actinobacteria to Proteobacteria, Acidobacteria, Firmicutes, Patescibacteria, and Actinobacteria; this indicates that soil conditioners play a significantly positive role in preventing and overcoming continuous cropping obstacles [22]. Using Chinese cabbage as an experimental material, a study revealed that adding soil conditioners increased the microbial richness indexes (Chao1 and ACE) and diversity indexes (Simpson and Shannon) of both fungi and bacteria. Moreover, the relative abundance of bacterial communities such as Actinobacteria, Chloroflexi, Gemmatimonadetes, and Bacteroidetes also increased. However, the relative abundance of Proteobacteria, Acidobacteria, Firmicutes, Nitrospirae, Verrucomicrobia, and Planctomycetes significantly decreased. This indicates that soil conditioners could effectively change the structure of the Chinese cabbage rhizosphere microbial community and optimize its health status [23].

This study explored the cucumber yield, fruit quality, and rhizosphere soil physicochemical properties and analyzed the soil microbial community using the Illumina MiSeq System to explore the effects of different volcanic slag ratios on cucumber yield, quality, and rhizosphere soil characteristics. The most suitable ratio of volcanic slag applied to cucumbers was selected, aiming to provide a theoretical reference for volcanic slag application in facility cultivation.

Through experiments, it was concluded that the appropriate amount of volcanic slag has a positive effect on the growth and development of cucumber plants, leaf photosynthetic performance, and fruit yield and quality and can improve the physical and chemical properties of cucumber rhizosphere soil and enhance soil enzyme activity. The recommended amount of volcanic slag in greenhouse cucumber production is 1000 kg/667 m^2^. Of course, there are still some limitations in this experiment. Whether the 1000 kg addition amount of 667 m^2^ is suitable for other soil types or crops still needs to be verified.

## 2. Results

### 2.1. Effects of Different Application Amounts of Volcanic Slag on Cucumber Yield

As shown in Table 1, the HS500, HS1000, and HS1500 treatments significantly increased the average weight of individual cucumber fruits by 5.57%, 7.91%, and 6.78%, respectively. In contrast, similar improvements were not observed under the HS2000 treatment. These results indicated that adding an appropriate amount of volcanic slag positively impacted the average weight of cucumbers. Based on the experimental conditions of this study, the HS1000 treatment resulted in the most significant increase in the average weight of individual cucumber fruits.

The cucumber yield increased by 6.86%, 17.67%, and 10.41% under the HS500, HS1000, and HS1500 treatments, respectively, compared with the control (CK) treatment. No significant change was observed under the HS2000 treatment compared with the CK treatment. This indicated that adding an appropriate amount of volcanic slag could increase cucumber yield, and the HS1000 treatment had the most significant promoting effect on increasing cucumber yield.

### 2.2. Effects of Different Application Amounts of Volcanic Slag on the Quality of Cucumber Fruits

As shown in Table 2, adding different amounts of volcanic slag positively impacted the quality of cucumber fruits. Different proportions of volcanic slag helped increase the soluble sugar content in cucumber fruits compared with the CK treatment. Specifically, the HS1000, HS1500, and HS500 treatments increased the soluble sugar content by 12.39%, 7.10%, and 5.51%, respectively, with HS1000 showing a prominent increase. The HS1000, HS1500, and HS2000 treatments increased the organic acid content by 34.98%, 26.46%, and 9.42%, respectively. HS500 and HS1000 increased the soluble protein content by 31.43% and 30.86%, while HS1500 and HS2000 increased the soluble protein content by 24% and 22.29%. Among them, HS500 and HS1000 increased the soluble protein content more obviously. It can be concluded that the appropriate amount of volcanic slag increased the soluble protein content in cucumber fruit. The appropriate amount of volcanic slag also positively impacted the vitamin C (Vc) content in cucumber fruits. The HS500, HS1000, HS1500, and HS2000 treatments all increased the Vc content by 10.42%, 17.57%, 14.62%, and 6.22%, respectively. Moreover, the HS500, HS1000, and HS1500 treatments increased the soluble solid content by 14.33%, 24.33%, and 19.00%, respectively, compared with the CK treatment.

The nitrate content in cucumber fruits decreased by 4.18%, 9.37%, 7.50%, and 5.41% under the HS500, HS1000, HS1500, and HS2000 treatments, respectively, compared with the CK treatment.

### 2.3. Effects of Different Application Amounts of Volcanic Slag on the Physicochemical Properties of Cucumber Rhizosphere Soil

As shown in Table 3, all treatments increased the pH of cucumber rhizosphere soil. The pH of cucumber rhizosphere soil increased by 0.74%, 2.87%, 5.11%, and 7% under the HS500, HS1000, HS1500, and HS2000 treatments, respectively, compared with the CK treatment. The results showed that adding volcanic slag could increase the pH of cucumber rhizosphere soil, and the phenomenon of pH increase was more pronounced on increasing the amount of volcanic slag. The HS2000 treatment showed the highest increase in the pH of cucumber rhizosphere soil. As shown in Table 3, the electrical conductivity of cucumber rhizosphere soil increased by 7.65%, 10.59%, 12.94%, and 15.29% under the HS500, HS1000, HS1500, and HS2000 treatments, respectively, compared with the CK treatment. These results indicated that the electrical conductivity of cucumber rhizosphere soil gradually increased with the increasing amount of volcanic slag added, and the HS2000 treatment showed the greatest increase in the electrical conductivity of cucumber rhizosphere soil.

As shown in Table 3, volcanic slag application was beneficial for an increase in total nitrogen (TN) in cucumber rhizosphere soil, increasing the total nitrogen content by 11.32%, 6.60%, 5.66%, and 1.89% under the HS1000, HS500, HS1500, and HS2000 treatments, respectively, compared with the CK treatment. Thus, volcanic slag application could increase the total nitrogen content in cucumber rhizosphere soil, and the HS1000 treatment exhibited the most obvious promotion effect on the total nitrogen content in cucumber rhizosphere soil. As shown in Table 3, volcanic slag application was beneficial for an increase in mineral nitrogen in cucumber rhizosphere soil, significantly increasing it by 32.49%, 16.56%, 13.07%, and 4.93% under the HS1000, HS1500, HS500, and HS2000 treatments, respectively, compared with the CK treatment. Thus, volcanic slag application could increase the mineral nitrogen content in cucumber rhizosphere soil, and the HS1000 treatment had the most obvious promotion effect on the mineral nitrogen content in cucumber rhizosphere soil.

As shown in Table 3, the ammonium nitrogen (NH4+-N) content in cucumber rhizosphere soil treated with HS500, HS1000, and HS1500 increased by 18.27%, 42.92%, and 18.53%, respectively, compared with the CK group. In contrast, the HS2000 treatment reduced the NH4+-N content in cucumber rhizosphere soil by 19.06%. This indicated that with the increase in the amount of volcanic slag soil conditioner, the NH4+-N content in the cucumber root soil first increased and then decreased. The effect of increasing the NH4+-N content in cucumber rhizosphere soil was the most significant under the HS1000 treatment, whereas the HS2000 treatment showed the greatest decrease in the NH4+-N content. As shown in Table 3, the HS1000 treatment group exhibited a significant increase of 43.97% in the organic matter content in cucumber rhizosphere soil compared with the CK group. The organic matter content in cucumber rhizosphere soil of other treatment groups also increased to varying degrees, with HS500, HS1500, and HS2000 treatment groups showing an increase in the content by 11.06%, 28.31%, and 36.44%, respectively. These data indicated that volcanic slag application could increase the organic matter content in cucumber rhizosphere soil, and the HS1000 treatment most prominently increased the organic matter content of the soil.

As shown in Table 3, all treatments increased the nitrate nitrogen content in the rhizosphere soil of cucumber compared with the CK treatment. The HS500, HS1000, HS1500, and HS2000 treatments achieved increases of 20.42%, 71.20%, 41.58%, and 26.50%, respectively. This indicated that volcanic slag application could enhance nitrate nitrogen content in cucumber rhizosphere soil, with the HS1000 treatment showing the most significant effect. Further, the HS500, HS1000, and HS1500 treatments increased the available phosphorus (AP) content in the cucumber rhizosphere soil by 53.54%, 60.96%, and 40.39%, respectively, compared with the CK treatment. However, the HS2000 treatment reduced the available phosphorus content by 11.74%. This result revealed that different amounts of volcanic slag had different effects on the available phosphorus content in cucumber rhizosphere soil. In this study, the HS1000 treatment had the most significant promoting effect on the available phosphorus content, whereas the HS2000 treatment exhibited the largest reduction in the available phosphorus content.

As shown in Table 3, the HS500, HS1000, HS1500, and HS2000 treatments significantly increased the available potassium (AK) content in cucumber rhizosphere soil by 8.10%, 23.45%, 14.2%, and 10.10%, respectively, compared with the CK treatment. This result indicated that volcanic slag application could effectively increase the available potassium content in cucumber rhizosphere soil, with HS1000 treatment showing the most significant promotion effect on the available potassium content in cucumber rhizosphere soil. Table 3 also shows that HS1000 and HS1500 treatments helped increase the water content in cucumber rhizosphere soil by 18.00% and 8.00%, respectively, compared with the CK treatment. In contrast, the changes in the water content under the HS500 and HS2000 treatments were not significant, and different amounts of volcanic slag had different effects on the water content in cucumber rhizosphere soil. The HS1000 treatment showed the most prominent promotion effect on the water content in cucumber rhizosphere soil.

The results of this study indicated that applying different amounts of volcanic slag improved the physical and chemical properties of cucumber rhizosphere soil, increased the nutrient content in the soil, and improved the soil quality. Among these, the total nitrogen, alkali-hydrolyzable nitrogen, NH4+-N, organic matter, nitrate nitrogen, available phosphorus, and available potassium contents in cucumber rhizosphere soil peaked under the HS1000 treatment. The NH4+-N and available phosphorus contents in cucumber rhizosphere soil were significantly lower under the HS2000 treatment compared with the CK treatment. The improvement effect was the best under the HS1000 treatment, whereas the HS2000 treatment showed a negative impact on the physical and chemical properties of cucumber rhizosphere soil.

### 2.4. Analysis of the Composition and Relative Abundance of Soil Bacteria in Cucumber Rhizosphere Soil with Different Application Amounts of Volcanic Slag

#### 2.4.1. Analysis of Soil Sequencing Results

**Figure 1:**
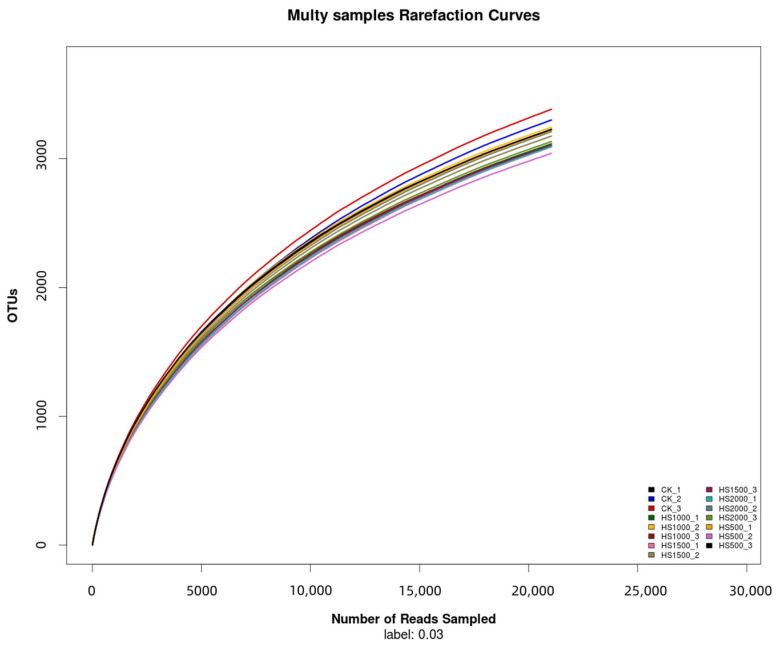
Sample dilution plot.

As shown in Figure 1, the dilution curve of the sample after processing had a flat trend, indicating that the sequencing data of the soil bacterial community in this experiment were reliable. With the increase in sequencing depth, the impact on the observed number and species of operational taxonomic units (OTUs) was minimal. Therefore, these data could objectively and accurately reflect the information of each treatment sample, providing a reliable data basis for further exploration of the bacterial community structure.

**Figure 2:**
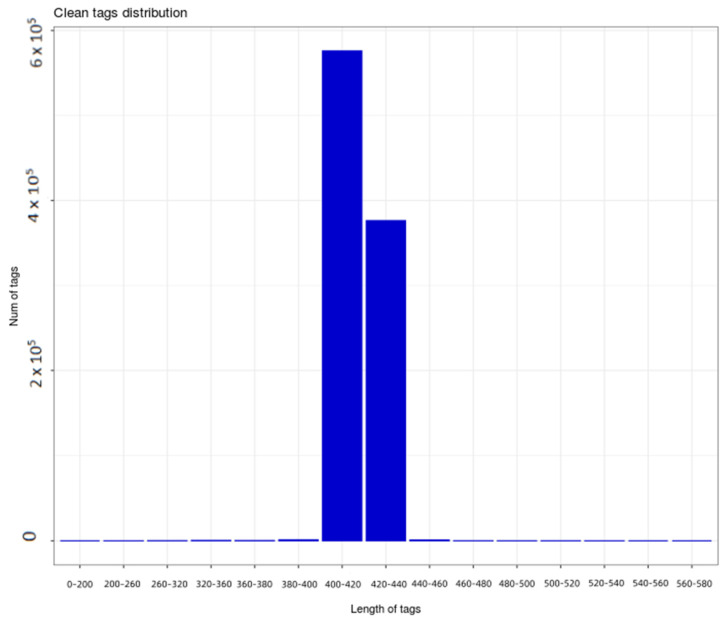
Distribution of quality sequences.

After filtering the sequencing results, 955,416 high-quality sequences were obtained by removing chimeras and short sequences (Figure 2). These sequences were mainly in the range of 400–440 bp, with most sequences (575,981) having lengths between 400 and 420 bp, followed by 376,297 sequences having lengths between 420 and 440 bp.

#### 2.4.2. Distribution of OTUs in the Cucumber Root Soil with Different Application Amounts of Volcanic Slag

**Figure 3:**
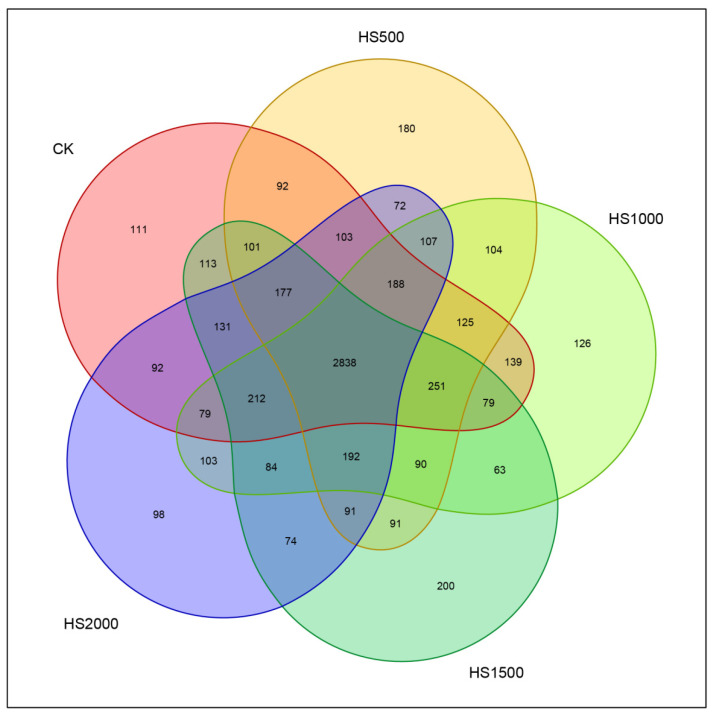
Distribution of bacterial OTUs using a Venn diagram.

Similarity analysis was conducted on all sequences at different similarity levels, and the OTU classification was based on clustering analysis of OTUs at a 97% similarity threshold. After rarefaction, an intuitive display of the distribution of OTU numbers and the overlap and unique features between groups was obtained using Venn diagrams (Figure 3). Among the volcanic slag samples with different addition amounts, 2838 shared OTUs were present. The CK treatment had 111 unique OTUs, whereas the HS500 treatment had 180 OTUs, representing a 62% increase compared with the CK treatment. The HS1000 treatment had 126 unique OTUs, representing a 13.5% increase compared with the CK treatment. The HS1500 treatment had 200 unique OTUs, an 80.1% increase. The HS2000 treatment had 98 unique OTUs, showing an 11.7% decrease compared with the CK treatment.

The CK treatment had 3215 OTUs in the cucumber root soil, whereas the HS500 treatment had 3217 OTUs, representing an increase of 0.06% compared with the CK treatment. The HS1000 treatment had 3101 OTUs in the cucumber root soil, representing a decrease of 3.55% compared with the CK treatment. The HS1500 treatment had 3213 OTUs in the cucumber root soil, representing a decrease of 0.06% compared with the CK treatment. The HS2000 treatment had 3146 OTUs in the cucumber root soil, representing a decrease of 2.15% compared with the CK treatment. These results indicated that the HS500, HS1000, and HS1500 treatments all increased the number of unique OTUs, whereas only the HS2000 treatment decreased the number of unique OTUs.

#### 2.4.3. Effects of Different Application Amounts of Volcanic Slag on the Alpha Diversity of Soil Bacteria in the Cucumber Root Soil

As shown in Table 4, when analyzing the alpha diversity indexes reflecting the richness and diversity of soil bacterial communities, it was observed that the HS1000 treatment increased the richness, ACE, and Shannon indexes by 5.18%, 5.12%, and 2.74% compared with the CK treatment. However, no significant differences in Simpson index and coverage were found between the groups. These results indicated that adding volcanic slag in moderation could effectively alter the richness and diversity of cucumber rhizosphere soil bacterial communities.

#### 2.4.4. Composition and Differential Analysis of Bacterial Communities in the Cucumber Root Soil with Different Application Amounts of Volcanic Slag

After applying varying amounts of volcanic slag to the soil around cucumber roots and clustering the 16srRNA annotations, it was found that the OTUs belonged to 43 phyla, 118 classes, 299 orders, 458 families, and 852 genera.

**Figure 4:**
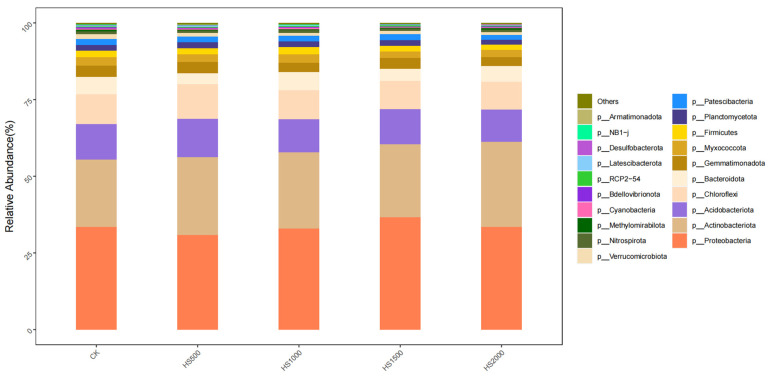
Comparison of relative abundances at the bacterial phylum level in the soil.

At the phylum level (Figure 4), the relative abundances of Proteobacteria (30.85–36.60%), Actinobacteriota (21.86–27.65%), Acidobacteriota (10.63–12.48%), Bacteroidota (3.58–6.00%), Gemmatimonadota (2.81–3.67%), Myxococcota (2.17–2.85%), Firmicutes (1.70–2.44%), Planctomycetota (1.61–2.00%), Patescibacteria (1.60–1.97%), and Verrucomicrobiota (0.93–1.56%) were all greater than 1%, making them the top 10 dominant phyla.

Among all treatments, Proteobacteria exhibited the highest relative abundance, with the highest percentage observed under the HS1500 treatment (36.60%) and the lowest under the HS500 treatment (30.85%). The abundance of Proteobacteria in each treatment was in the order HS1500 > CK > HS2000 > HS1000 > HS500. The relative abundance of Actinobacteriota was the highest under the HS2000 treatment with a percentage of 27.65%, and the lowest under the CK treatment with a percentage of 21.86%. The abundance of Actinobacteriota in each treatment was in the order HS2000 > HS500 > HS1000 > HS1500 > CK. The relative abundance of Acidobacteria was the highest under the HS500 treatment with a percentage of 12.48%, and the lowest under the HS2000 treatment with a percentage of 10.63%. The order of abundance of Acidobacteria in each treatment was HS500 > CK > HS1500 > HS1000 > HS2000. The relative abundance of Planctomycetes was the highest under the HS1000 treatment with a percentage of 6%, and the lowest under the HS500 treatment with a percentage of 3.58%. The abundance of Planctomycetes in each treatment was in the order HS1000 > CK > HS1500 > HS2000 > HS500. The relative abundance of Gemmatimonadetes was the highest under the HS500 treatment with a percentage of 3.67%, and the lowest under the HS2000 treatment with a percentage of 2.81%. The abundance of Gemmatimonadetes in each treatment was in the order HS500 > CK > HS1500 > HS1000 > HS2000. The relative abundance of Verrucomicrobia was the highest under the CK treatment with a percentage of 2.85%, and the lowest under the HS1500 treatment, with a percentage of 2.17%. The abundance of Verrucomicrobia in each treatment was in the order CK > HS1000 > HS500 > HS2000 > HS1500. The relative abundance of Firmicutes was the highest under the HS1000 treatment with a percentage of 2.44%, and the lowest under the HS2000 treatment with a percentage of 1.70%. The abundance of Firmicutes in each treatment was in the order of HS1000 > HS500 > CK > HS1500 > HS2000. The relative abundance of Ascomycota was the highest under the HS500 treatment with a percentage of 2%, and the lowest under the HS2000 treatment with a percentage of 1.61%. The abundance of Ascomycota in each treatment was in the order HS500 > CK > HS1000 > HS1500 > HS2000. The relative abundance of Basidiomycota was the highest under the HS1500 treatment with a percentage of 1.97%, and the lowest under the CK treatment with a percentage of 1.56%. The abundance of Basidiomycota in each treatment was in the order HS1500 > CK > HS1000 > HS500 > HS2000.

This study of the bacterial community at the phylum level in the cucumber rhizosphere after applying different amounts of volcanic slag showed no significant difference in the relative abundance of dominant phyla among the treatments. However, variations in the proportion and species of dominant phyla were observed across different treatments. Therefore, we further analyzed the composition of the bacterial community structure at the genus level.

#### 2.4.5. Impact of Environmental Factors on the Distribution of Bacterial Communities in Cucumber Rhizosphere Soil

**Figure 5:**
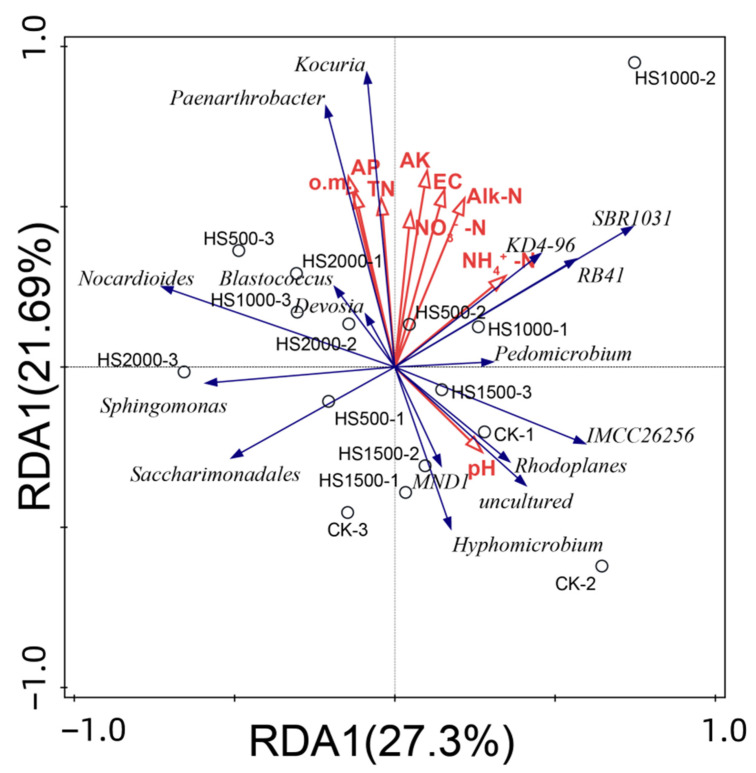
Redundancy analysis diagram. Note: The points represent the matrix sample; the red arrows represent the physicochemical properties of the matrix; and the blue arrows represent the matrix microbiota. An acute angle between the influencing factors (factors and samples) indicates a positive correlation between the two factors, and an obtuse angle indicates a negative correlation. The longer the arrow, the greater the effect of the factor. available potassium, Available potassium; Alk-N, alkaline nitrogen; available phosphorus, available phosphorus; EC, electrical conductivity; NH4+-N, ammonium nitrogen; NO_3_–N, nitrate nitrogen; o.m., organic matter; pH, acidity and alkalinity; total nitrogen, total nitrogen; VWC, volumetric water content.

The relative abundance was analyzed concerning soil physicochemical properties based on the dominant bacterial taxa at the genus level in cucumber rhizosphere soil after different volcanic slag treatments (Figure 5). The figure shows that 9 soil physicochemical indicators and 16 bacterial genera collectively accounted for 27.3% and 21.69% of the redundancy analysis (RDA) total variation, respectively, totaling 48.99%. This indicated that the contribution values of the first two axes could better explain the influence of various environmental factors on the soil bacterial community structure. The soil physicochemical indicators were mainly concentrated in the first and second quadrants, where Paenarthrobacter, SBR1031, and Kocuria were positively correlated with eight soil physicochemical indicators, such as available potassium and total nitrogen, and negatively correlated with pH. Nocardioides was negatively correlated with alkaline nitrogen, pH, and NH4+-N, and positively correlated with six soil physicochemical indicators, such as available potassium and total nitrogen. Hyphomicrobium and IMCC26256 were positively correlated with pH in the third and fourth quadrants and negatively correlated with eight soil physicochemical indicators, such as available potassium and total nitrogen. The longest radii of available phosphorus, available potassium, and NH4+-N in the soil had the highest cumulative explanatory power, which was the main influencing factor driving changes in the bacterial community at the genus level.

## 3. Discussion

Volcanic slag is primarily composed of volcanic glass and a smaller proportion of zeolite (such as clinoptilolite) [24], containing minerals such as iron, magnesium, and silicon [25], as well as trace elements such as nickel and cadmium [26]. Volcanic slag has good adsorption capacity, providing new possibilities for the growth and development of crops in soil. Plants use their root systems to directly absorb nutrients from the soil, and the health of the roots affects the water and nutrient supply to the aboveground and underground parts of the plant. A plant’s root system is essential not only for absorbing water and various nutrients required to nourish the aboveground parts but also for ensuring the adequacy of nutrients needed for the roots to maintain their life activities [27]. Regarding cucumber yield, different application amounts of volcanic slag treatment did not significantly affect the number of fruits per plant but significantly increased the yield by increasing the average weight of individual fruits. This is because volcanic slag application promoted the growth of the nutritional organs of cucumber roots, stems, and leaves, allowing the absorption of more nutrients from the soil. This led to better fruit development and a higher yield of cucumbers.

This study also found that volcanic slag applied to the root zone of cucumbers significantly increased the soluble sugar, organic acid, soluble protein, and vitamin C contents in cucumber fruits and significantly reduced the nitrate content. Treatment with HS500, HS1000, and HS1500 also significantly increased the soluble solid content in cucumbers. The results indicated that volcanic slag application significantly enhanced the antioxidant capacity, photosynthetic pigments, and photosynthesis of cucumber leaves, promoting the accumulation and distribution of dry matter in cucumber plants and ultimately improving the quality of cucumber fruits. Soluble proteins served as key osmotic regulators crucial for the growth of cucumber plants. At the same time, vitamin C served as a typical antioxidant substance in plants, reflecting the optimization of cucumber’s environmental adaptability after adding volcanic slag. The combined effects of these factors not only improved the antioxidant capacity of cucumbers but also enhanced the nutritional value and taste of cucumber fruits. This finding was consistent with the research results of Xu Ning [28] and Dong Yu [29] on the quality of cucumber fruits, but the mechanism needs further exploration.

Overall, considering the yield and fruit quality of cucumbers, the HS1000 treatment was the most effective, showing the best improvement under the conditions of this experiment.

The physical and chemical properties of soil are crucial indicators of its health status, reflecting the nutrient content in the soil and the overall soil quality [30,31]. In this study, we observed that different proportions of volcanic slag significantly impacted the cucumber rhizosphere soil environment. These treatments increased the pH of cucumber rhizosphere soil compared with the CK treatment, and the effect was enhanced by increasing the amount of volcanic slag added. The increase in soil pH was due to the alkaline rock fragments carried by the volcanic slag [32]. All treatments significantly increased the electrical conductivity, total nitrogen, alkali-hydrolyzable nitrogen, organic matter, nitrate nitrogen, and available potassium content of cucumber rhizosphere soil compared with the CK treatment. The appropriate amount of volcanic slag treatment also had a good promoting effect on NH4+-N, available phosphorus, and water content in cucumber rhizosphere soil. In contrast, the HS2000 treatment reduced the NH4+-N and available phosphorus contents in cucumber rhizosphere soil compared with the CK treatment. The improved effect was due to the good adsorption of volcanic slag, which aggregated fine soil and clay particles into soil aggregates, coagulated soil colloids, promoted the formation of soil aggregates, and enhanced the soil’s water retention capacity and permeability [33,34]. The irregular arrangement of volcanic slag particles created large pores, enhancing the soil’s ion exchange capacity [35], nitrogen fixation, denitrification, and phosphorus solubilization abilities [36]. The mineral-rich volcanic slag also enriched the soil nutrients. However, applying a high amount of volcanic slag led to secondary salinization of cucumber rhizosphere soil, causing complex salt reactions and leaching of bases, negatively impacting cucumber rhizosphere soil.

Microorganisms play an essential role as decomposers in the natural world, acting as important participants in material cycling in the soil. Rhizosphere microorganisms are the closest to plants and are crucial in soil nutrient cycling, which is significant for maintaining soil fertility [37]. The Illumina MiSeq System high-throughput sequencing technology was used in this study to explore the changes in bacterial community structure and diversity in cucumber rhizosphere soil with different application rates of volcanic slag soil conditioner.

The alpha diversity analysis reflects the richness and diversity of soil bacterial communities. The richness and ACE indexes explain the community richness of soil bacteria [38], whereas the Shannon [39] and Simpson indexes can explain the community diversity of soil bacteria. This experiment found that only the HS1000 treatment significantly increased the richness, ACE, and Shannon indexes, whereas the Simpson index and coverage did not show significant changes between groups. The dominant phyla in each treatment were Proteobacteria, Actinobacteria, and Acidobacteria, among 10 phyla, which was consistent with the findings of Xu Hongli [40] and Hu Yun [41] on cucumbers.

Adding volcanic slag promoted the increase in beneficial bacterial community abundance in cucumber rhizosphere soil, enhancing the denitrification, phosphorus solubilization, and nitrogen fixation abilities of the soil, while also alleviating autotoxicity and enhancing biological CK capabilities. It created suitable soil conditions and growth environments for cucumber growth, improving cucumber rhizosphere soil, which is reflected in the increase in soil physicochemical properties and cucumber agronomic traits. The RDA of soil physicochemical properties and 15 bacterial genera of interest revealed that the available phosphorus, available potassium, and NH4+-N contents in the soil were the main influencing factors of the bacterial community in cucumber rhizosphere soil, consistent with the findings of Zhang Fan [20], Sun Shijun [42], and Du Juan [43] on vegetable crops.

## 4. Experimental Materials and Methods

### 4.1. Materials

The cucumber variety “Jiza No. 4” used in this study was provided by Jilin Vegetable and Flower Science Research Institute.

Volcanic slag was provided by Jilin Jiujin Agricultural Science and Technology Co., Ltd. (Jilin, China). The pH value of the volcanic slag was 7.34, and the organic matter, available phosphorus, available potassium, and total nitrogen contents were 55.70%, 5.6 mg/kg^−1^, 159.8 mg/kg^−1^, and 0.011 g/100 g^−1^, respectively. The experiment was conducted in the greenhouse of the Horticulture College Teaching and Experimental Base of Jilin Agricultural University. The original soil bulk density was 0.92 g/cm^−3^, the pH was 6.88, the organic matter content was 60.6 g/kg^−1^, the alkali nitrogen content was 110.74 mg/kg^−1^, the available phosphorus content was 117.88 mg/kg^−1^, and the available potassium content was 48.62 mg/kg^−1^.

### 4.2. Methods

The experiment started in early December 2022 at the teaching experimental base greenhouse of the College of Horticulture, Jilin Agricultural University, and ended near the end of February 2024. The experiment used trough cultivation with four experimental treatments and one CK treatment. A total of 5 treatments (Table 5) The amount of volcanic slag added (kg/667 sq.m) in the cultivation trough was 500 kg (HS500), 1000 kg (HS1000), 1500 kg (HS1500), and 2000 kg (HS2000), with 0 kg as the CK treatment. Volcanic slag and soil were evenly stirred and fully mixed in the soil at 30 cm. The row spacing was 35 cm × 55 cm, and each treatment was a cultivation tank. The area of the cultivation tank was 3.22 m^2^, and each repetition was 54 plants with a total of three repetitions, and the randomized complete block design was carried out.

#### 4.2.1. Determination of Cucumber Growth Indicators and Yield

In each treatment, three plants were randomly selected for fixed observation. After absorbing the moisture from the plant roots using filter paper, the fresh weight was measured using an electronic scale. For dry weight, the roots were dried using a dryer and measured using an electronic scale.

Each plant was weighed and counted individually during fruit picking, and the quantity and yield of the results were recorded. The total yield was calculated based on the number of plants and their individual yields. In each treatment, 10 cucumber fruits free from mechanical damage, pests, and diseases were randomly picked, and their weights were measured using an electronic weighing method.

#### 4.2.2. Determination of Cucumber Fruit Quality

Fifteen cucumber fruits with uniform growth were picked from different treatment groups, placed in a thermos, and directly sent to the laboratory for testing various indicators. The soluble total sugar content was determined using the anthrone colorimetric method [44], the Vc content using the 2,6-dichlorophenol indophenol titration method [44], the acid content using the acid–base titration method [44], the soluble solid content using a liquid concentration meter (PAL-1 type) [44], and the nitrate content using [44].

#### 4.2.3. Collection of Soil Samples

The “S”-shaped random sampling method was used for collecting soil samples. The samples were collected from the rhizosphere soil area of cucumber plants. The samples were passed through a 2 mm sieve to remove roots and weeds and transferred to the laboratory. Some samples were air-dried at room temperature for evaluating soil physical and chemical properties, and the other samples were frozen at −80 °C for microbial quantitative polymerase chain reaction (PCR) analysis and high-throughput sequencing.

#### 4.2.4. Determination of Soil Physical and Chemical Properties

The organic matter content in the soil was determined using the potassium dichromate volumetric method [45]. The alkali diffusion method was used to determine the alkali nitrogen, nitrate nitrogen, and NH4+-N contents [45]. The available phosphorus and available potassium contents were determined using the sodium bicarbonate leaching spectrophotometric method [45] and the ammonium acetate leaching flame photometer method, respectively [45]. The pH and electrical conductivity were determined using the potentiometric method. A soil-water mixture at a 1:5 ratio was prepared and allowed to stand for 30 min before analysis using a potentiometer [45]. The total nitrogen content in the soil was determined using the Kjeldahl method [45].

##### Soil Bacteria Sequencing Analysis with DNA Extraction

Five processed substrate samples were collected and sent to Beijing Ovisen Genetic Technology Co., Ltd. (Beijing, China) for soil microbial DNA extraction using the Omega Stool DNA Kit (MoBio Laboratories, Carlsbad, CA, USA) for total DNA extraction. The extracted DNA was tested for quality and concentration using spectrophotometry. The samples that passed quality control were stored at −20 °C for future experimental use.

##### PCR Amplification and Purification, and MiSeq Sequencing

The bacterial 16S rRNA gene V3–V4 region was amplified using primers 338F (5′-ACTCCTACGGGAGGCAGCAG-3′) and 806 (5′-GGACTACNNGGG-TATCTAAT-3′). Eight base pair tags were added to the 5′ end of the upstream and downstream primers for sample identification. The PCR reaction system (total volume 25 μL) included 12.5 μL of 2xTaq Plus Master Mix, 3 μL of bovine serum albumin (2 ng/μL), 1 μL of forward primer (5 μM), 1 μL of reverse primer (5 μM), 2 μL of DNA (total amount of added DNA was 30 ng), and 5.5 μL of ddH2O to reach a final volume of 25 μL. The reaction parameters were as follows: initial denaturation at 95 °C for 5 min; denaturation at 94 °C for 30 s, annealing at 50 °C for 30 s, and extension at 72 °C for 60 s, for 30 cycles; and final extension at 72 °C for 7 min. The PCR products were analyzed using 1% agarose gel electrophoresis to determine the size of the amplified bands, followed by purification using the Agencourt AMPure XP nucleic acid purification kit.

Using the Illumina MiSeq PE300 high-throughput sequencing platform for paired-end sequencing, the sequencing raw data were uploaded to the SRA database of NCBI.

##### Statistical Analysis

The QIIME1 (v.1.8.0) software was used to split samples according to Barcode sequences, and Pear (v0.9.6) software was used to filter the data. Fuzzy bases, sequence mismatches, and scores below 20 were deleted. The minimum coverage value for splicing was 10 bp, with a mismatch rate of 0.1. Fragments larger than 230 bp were selected using Vsearch (v2.7.1) software and matched and removed using the UCHIME method to achieve 97% sequence similarity. All samples were normalized to 46,610 sequences to ensure a high coverage range across all. The RDP Classifier algorithm was used to compare sequences with the SILVA 128 database, with a confidence level set at 70%, to obtain the necessary species information for each OTU. Based on these results, a bar graph analysis of species composition was conducted using R (v3.6.0) software.

## 5. Data Processing

Data processing was conducted using SPSS (27.0) and Origin2021 software, with significance analysis using LSD and Duncan methods (*p* < 0.05). High-quality sequences were clustered into OTUs using the UPARSE algorithm in Vsearch (v2.7.1) software, and alpha diversity analysis (including Shannon, Simpson, ACE Coverage, and Chao1 indexes) was performed using QIIME1 (v1.8.0) software. Shannon: H = −∑(Pi)(ln Pi), Simpson: D = 1 − ∑(Ni/N)^2^, ACE: Sabund: high abundance species (abundance > 10); Srare: rare species (abundance ≤ 10 and not 0); f: Species with abundance = 1 (singleton), Coverage: C = 1 − n1/N, Chao1 = Sobs + n1(n1 − 1)/2(n2 + 1). Species composition bar graph analysis was conducted using R (v3.6.0) software. Clustering heat maps were generated based on the weighted UniFrac distance using R (v3.6.0) software.

## 6. Conclusions

1. The appropriate addition of volcanic slag positively impacted the growth and development of cucumber plants, photosynthetic performance of leaves, fruit yield, and quality. All treatments promoted the growth of cucumber plants and improved the photosynthetic characteristics of cucumber leaves. The cucumber yield significantly increased by 24.28% under the HS1000 treatment. The soluble sugar, Vc, and soluble solid contents increased by 12.39%, 17.57%, and 24.33%, respectively, whereas the nitrate content decreased by 9.37% under the HS1000 treatment compared with the CK treatment.
2. The appropriate amount of volcanic slag could improve the physical and chemical properties of cucumber rhizosphere soil. The electrical conductivity and organic matter, alkali nitrogen, nitrate nitrogen, and available potassium contents significantly increased under each treatment, whereas total nitrogen, available phosphorus, NH4+-N, and moisture contents increased to varying degrees. The pH of the soil increased with the increase in the amount of volcanic slag applied.
3. Through Illumina MiSeq sequencing, clustering at a 97% similarity level, and analysis of alpha diversity and RDA, available phosphorus, available potassium, and NH4+-N were identified as the main influencing factors of changes in the bacterial community structure in cucumber rhizosphere soil.

In this study, a comprehensive evaluation under the conditions discussed recommends adding 1000 kg/667 m^2^ of volcanic slag as the optimal amount for cucumber production facilities, corresponding to the HS1000 treatment.

## Figures and Tables

**Table 1:** Effects of different application amounts of cinders on cucumber production.

Treatment	Average Ear Weight (g)	Yield (kg/ha)
CK	195.46 ± 3.08 c	42,759.44 ± 114.15 c
HS500	206.35 ± 1.8 b	45,693.13 ± 103.89 b
HS1000	210.93 ± 1.61 a	50,057.37 ± 116.36 a
HS1500	208.72 ± 3.18 ab	47,211.19 ± 47.54 ab
HS2000	195.88 ± 0.68 c	42,780.11 ± 87.56 c

Note: Different letters in the same column indicate significant differences (*p* < 0.05). Three repeats were used; the same applies below.

**Table 2:** Effects of different application amounts of volcanic slag on the quality of cucumber fruits.

Treatment	Soluble Sugar (%)	Titratable Acid (%)	Soluble Protein (mg/g^−1^)	Vitamin C (mg/100 g^−1^)	Soluble Solid Content (%)	Nitrate (μg·g^−1^ FW)
CK	5.81 ± 0.13 c	2.23 ± 0.01 d	1.75 ± 0.04 c	6.43 ± 1.13 d	3.00 ± 0.11 c	202.72 ± 0.21 a
HS500	6.16 ± 0.06 b	2.42 ± 0.01 c	2.30 ± 0.12 a	7.11 ± 2.26 c	3.43 ± 0.07 b	194.24 ± 0.17 b
HS1000	6.53 ± 0.06 a	3.01 ± 0.01 a	2.29 ± 0.04 a	7.56 ± 2.83 a	3.73 ± 0.17 a	183.73 ± 0.21 d
HS1500	6.22 ± 0.04 b	2.82 ± 0.01 b	2.17 ± 0.01 ab	7.37 ± 1.17 b	3.57 ± 0.07 b	187.51 ± 0.39 c
HS2000	6.13 ± 0.02 b	2.44 ± 0.03 c	2.14 ± 0.03 b	6.83 ± 1.03 c	3.10 ± 0.11 c	191.75 ± 0.46 b

Note: Different letters in the same column indicate significant differences (*p* < 0.05).

**Table 3:** Effects of different application amounts of cinders on the physical and chemical properties of the cucumber rhizosphere soil.

Treatment	pH	Electrical Conductivity (ms/cm^−1^)	Total Nitrogen (g/kg^−1^)	Alkaline-Hydrolyzable Nitrogen (mg/kg^−1^)	Ammonium Nitrogen (mg/kg^−1^)	Organic Matter (g/kg^−1^)	Nitrate Nitrogen (mg/kg^−1^)	Quick-Acting Phosphorus (mg/kg^−1^)	Quick-Acting Potassium (mg/kg^−1^)	Moisture Content (%)
CK	6.806 d	1.67	1.05	156.79	11.43	94.20	25.66	132.26	153.42	1.93
HS500	6.881 d	1.78	1.11	177.66	13.52	104.63	29.98	234.30	165.45	1.91
HS1000	6.999 c	1.81	1.22	207.01	16.78	135.62	44.11	245.62	189.19	2.11
HS1500	7.003 b	1.83	1.09	182.54	13.66	120.87	36.35	214.22	175.29	2.00
HS2000	7.193 a	1.87	1.06	164.68	9.35	128.53	32.18	134.11	168.32	1.93

Note: Different letters in the same column indicate significant differences (*p* < 0.05).

**Table 4:** Alpha diversity indexes of cucumber rhizosphere soil with different application amounts of volcanic slag.

Treatment	Chao Index	ACE Index	Shannon Index	Simpson Index	Coverage (%)
CK	4473.58 ± 62.71 b	4549.91 ± 36.81 b	9.84 ± 0.04 b	1.00 ± 0.01 a	94 a
HS500	4508.98 ± 27.89 ab	4613.49 ± 17.91 ab	9.97 ± 0.01 ab	1.00 ± 0.01 a	94 a
HS1000	4705.34 ± 57.51 a	4786.83 ± 41.89 a	10.11 ± 0.02 a	0.99 ± 0.02 a	94 a
HS1500	4479.91 ± 43.56 ab	4603.72 ± 37.91 ab	9.93 ± 0.03 ab	1.00 ± 0.02 a	94 a
HS2000	4479.58 ± 47.59 ab	4581.04 ± 26.51 ab	9.93 ± 0.02 ab	1.00 ± 0.02 a	94 a

Note: Different letters in the same column indicate significant differences (*p* < 0.05).

**Table 5:** Volcanic slag application rate.

Process Name	Volcanic Slag Addition (kg/667 sq.m)
HS0 (CK)	0
HS500	500
HS1000	1000
HS1500	1500
HS2000	2000

## Data Availability

The original contributions presented in this study are included in the article. Further inquiries can be directed to the corresponding author(s).
